# CircRNA May Not Be “Circular”

**DOI:** 10.3389/fgene.2021.633750

**Published:** 2021-02-19

**Authors:** Handong Sun, Zijuan Wu, Ming Liu, Liang Yu, Jianyong Li, Jinwen Zhang, Xiangming Ding, Hui Jin

**Affiliations:** ^1^Department of Breast Surgery, The First Affiliated Hospital of Nanjing Medical University, Nanjing, China; ^2^Department of Hematology, The First Affiliated Hospital of Nanjing Medical University, Jiangsu Province Hospital, Nanjing, China; ^3^Key Laboratory of Hematology of Nanjing Medical University, Nanjing, China; ^4^Collaborative Innovation Center for Cancer Personalized Medicine, Nanjing, China; ^5^Guangzhou Geneseed Biotech Co., Ltd., Guangzhou, China; ^6^Department of Hematology, The Affiliated Huaian No.1 People’s Hospital of Nanjing Medical University, Huai’an, China; ^7^Department of Bioinformatics, ATCGene Inc., Guangzhou, China

**Keywords:** circular RNA, bio-informatics, biological phenomena, molecular conformation, hypothesis and theory

## Abstract

Circular RNA (circRNA) is a novel regulatory non-coding RNA and participates in diverse physiological and pathological processes. However, the structures and molecular mechanisms of circRNAs remain unclear. In this study, taking advantage of openly databases and bioinformatics analysis, we observed lots of internal complementary base-pairing sequences (ICBPS) existed in plenty of circRNAs, especially in extremely long circRNAs (el-circRNAs, > 5,000 nt). The result indicated that circRNA may not be a simple circular structure. In addition, we put forward the hypothesis of “open-close effect” in the transition for specific circRNA from normal state to morbid state. Taken together, our results not only expand the knowledge of circRNAs, but also highlight the potential molecular mechanism of circRNAs.

## Introduction

CircRNA, a group of endogenous non-coding RNAs, has long been characterized as a single strand covalently closed continuous loop without 5′–3′ polarity and a polyadenylated tail (Hansen et al., 2013). The expression of circRNAs has spatio-temporal specificity, and they may exhibit distinct expression patterns in different diseases or at different stages. It has been recently recommended that cirRNAs have an essential role in regulating genes expression by functioning as a translational regulator, RNA binding protein (RBP) sponge, and microRNA (miRNA) sponge (Memczak et al., 2013; Abbaszadeh-Goudarzi et al., 2020; Mirzaei and Hamblin, 2020). The most thoroughly studied function of circRNA is that it serves as competitive endogenous RNAs (ceRNAs). And studies have demonstrated their activity as miRNA sponges as well as protein sponges (Huang et al., 2020). Though accumulating evidence reveals that circRNAs could exert vital biological functions and serve as novel biomarkers as well as providing promising therapeutic approaches for various human diseases (Borran et al., 2020; Naeli et al., 2020; Razavi et al., 2020), much has not yet to be elucidated about their molecular mechanisms. Chen et al. (Liu C.X. et al., 2019) have recently exhibited the secondary structures of circRNAs and the structures are stable and formed from static process. However, the structures of circRNAs, especially their dynamic process are still unclear. In addition, current research of el-circRNAs is scarce. Here, circRNAs with two internal completely complementary regions (≥10 nt) are selected. And the sequences were acquired and regarded as candidate ICBPS. Existence of ICBPS may contribute to the difficulty of exploring el-circRNAs. To better figure out the concepts and theory put forward in the research, an el-circRNA (hsa_circ_0000527) with high complementary ratio (CR) and long maximum length (maxLen) of the ICBPS was taken as an example. Here, we propose a new hypothesis that circRNAs may not be a single strand continuous loop, and they probably have double-strand structure, which may be dynamically reversible and have impact on their mechanism and functions.

## Results

### Characteristics of ICBPS in circRNA

**FIGURE 1:**
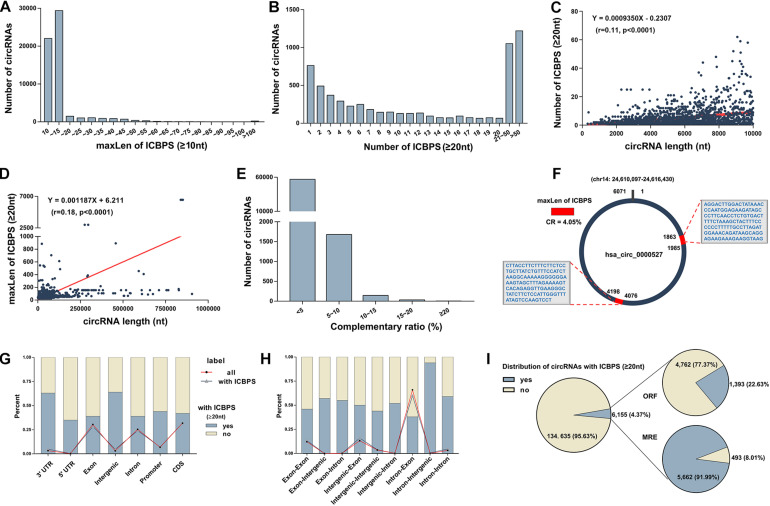
Characteristics of circRNAs **(A)** The number of circRNAs with different maxLen of ICBPS (≥10 nt). **(B)** The number of circRNAs with different amount of ICBPS. **(C)** The correlation analysis between the number of ICBPS (≥20 nt) and the total length of circRNAs (≤10,000 nt). **(D)** The correlation analysis between the maxLen of ICBPS (≥20 nt) and the total length of circRNAs. **(E)** The number of circRNAs with different complementary ratio. **(F)** Hsa_circ_0000527 was taken as an example. **(G)** The percentage of circRNAs (with ICBPS ≥ 20 nt or not) that transcribed from different regions of their parental gene. The polyline graph shows the number distribution of circRNAs in different types. **(H)** The percentage of circRNAs (with ICBPS ≥ 20 nt or not) with different components originated from the parental genes. The polyline graph shows the number distribution of circRNAs in different types. **(I)** The distribution of circRNAs with ICBPS ≥ 20 nt, and the distribution of these circRNAs with ORF/MRE at the same time.

In our previous study, we detected circRNAs’ expression in human plasma using circRNA microarray (GEO accession number: GSE131469) (Wu et al., 2019). Surprisingly, we discovered multiple of internal complementary base-pairing sequences (ICBPS) existed in circRNAs, especially in extremely long circRNAs (el-circRNAs,>5,000 nt). Given to the limited number of circRNAs in the microarray, we used human circRNA database^1^ (Liu M. et al., 2019) in the following research. Based on the bioinformatics analysis of this database that containing about 140,790 circRNAs, we got similar results. For most circRNAs, the maximum length (maxLen) of the ICBPS is under 15 or even 10 nt (Figure 1A). Next in this study, we emphasized on the analysis of 6,155 circRNAs (4.37% of total circRNAs) with ICBPS ≥ 20 nt, most of which contain more than one pair of ICBPS. The number of circRNAs with different amounts of ICBPS was analyzed statistically, and there are over 2,000 circRNAs containing more than 20 pairs of ICBPS (Figure 1B). Through bioinformatics analysis we found that the 6,155 circRNAs contain 58,995 ICBPS in total, and for 90% circRNAs, the median length (medianLen) of the ICBPS is between 20 and 31 nt (Supplementary Figure 1a). Also, the number and maxLen of ICBPS were closely correlated with the total length of circRNAs (circLen) (Figure 1C,D and Supplementary Figure 1b).

Next, we defined the concept of complementary ratio

$$CR,CR=\frac{\left(maxLenofICBPS\right)\times 2}{circLen}\times 100\%.$$

A higher CR would indicate a greater probability of internal base pairing in a circRNA. However, we found that CR of most circRNAs is under 10% or even 5%. And for circRNAs with higher CR, their circLens are all under 200 nt (Figure 1E, Supplementary Table 1). To better explain and clarified these concepts, here we include a detailed example. Hsa_circ_0000527, an el-circRNA with the circLen of 6,071 nt originating from exon 24 of chromodomain 14 was list as an example. Containing three pairs of ICBPS (≥20 nt), the maxLen of hsa_circ_0000527 is 123 nt, thus its CR is 4.05% (Figure 1F, Supplementary Date 1).

To characterize the ICBPS of circRNAs, we analyzed the locations and regions of parental genes. We found that for circRNAs that derived from 3′ untranslated regions (3′-UTR) of parental genes, about 60% of them contain ICBPS ≥ 20 nt (Figure 1G). Similar statistics analysis was conducted according to different chromosome origins as well as different components originated from the parental genes (Supplementary Figure 1c and Figure 1H).

Studies have confirmed that, functioning as ceRNA, circRNAs can competitively sponge microRNAs (miRNAs) through miRNA recognition elements (MREs) (Piwecka et al., 2017; Das et al., 2020). Analyzing the 206 circRNAs whose maxLen of ICBPS > 100 nt, we surprisingly observed that about 64% circRNAs have overlap between ICBPS and MREs (Supplementary Figure 1d). Meanwhile, circRNAs that containing internal ribozyme entry site (IRES) have the potential to translate proteins, which can be predicted through bioinformatics analysis of its open reading frame (ORF) (Legnini et al., 2017). Based on the analysis of circBank database, there are 6,155 circRNAs contain ICBPS ≥ 20 nt, and about 23/92% of them have overlap with ORF/MRE at the same time (Figure 1I).

### Possible Structures and Molecular Mechanisms of circRNAs

**FIGURE 2:**
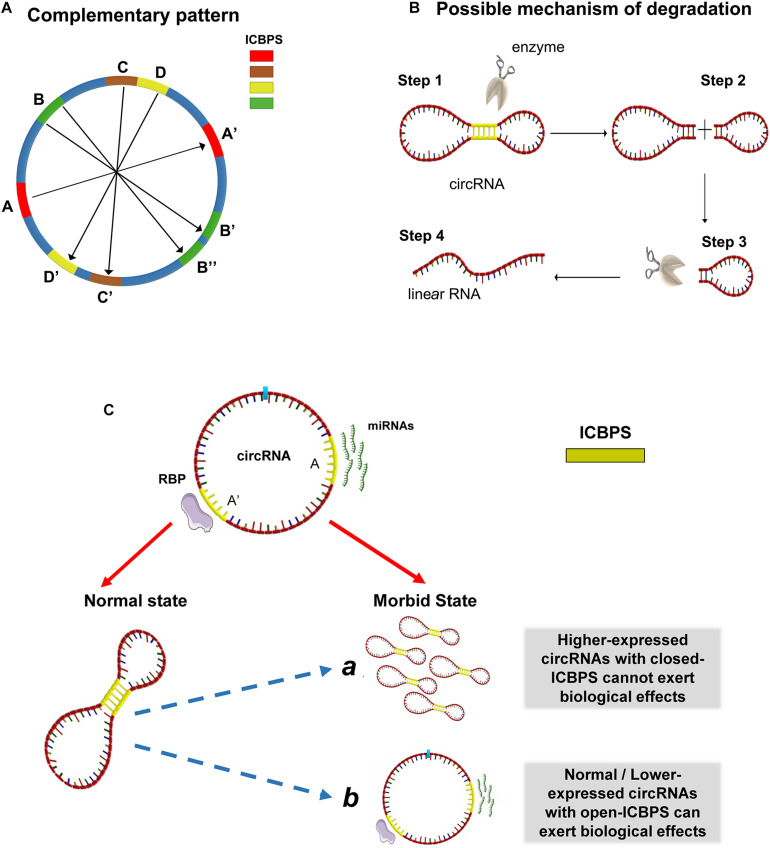
Possible structures and molecular mechanisms of circRNAs. **(A)** The complementary patterns of ICBPS in circRNA. **(B)** Possible mechanism of circRNA degradation due to the presence of double-strand structure. **(C)** The “open-close effect” in the transition for specific circRNA from normal state to morbid state. ICBPS was sequences with two internal completely complementary regions (≥10 nt). The existence of ICBPS affected RBP sponging and miRNA sponging.

Synthesizing the above analytical results, we conject that circRNA may not be a simple circular structure. It probably contains double-strand structure internally because of the presence of ICBPS (shown as A and A’ in Figure 2A). Special situations can exist. For example, there may be one segment of ICBPS that can be complementary paired with multiple ICBPS (shown as B and B’/B” in Figure 2A). Or for one continuous sequence, it may have different complementary sequences that set close together or overlap on the same RNA chain (shown as C, D and C’, D’ in Figure 2A). Thus, the “open” or “close” state of the double-strand structures in circRNAs is a sophisticated dynamic process. The formation of this structure makes circRNAs compressed in space, which may help circRNAs bond firmly with RBPs and thus facilitate themselves being exported into the cytoplasm from nucleus. The dynamic process is shown in video 1. However, the formation process of this structure might be reversible. After “escaping” from cell nucleus, circRNAs quickly switch from the “close” state to “open.” It is well-known that circRNAs are highly stable, but the degradation mechanism has not been clarified yet. The double-strand structure in circRNA may make them easier to be degraded by relevant enzymes which can probably explain how cells eliminate circRNAs (Figure 2B). The process may be regulated by micro-environment or other internal factors such as the length of ICBPS, the binding free energy, the distance between pairing fragments, the secondary structure of RNA, or relevant RNA modification like N6-methyladenosine (m6A), etc. At the same time, it might be an important way to regulate the circRNAs’ degradation, translation and adsorption of miRNAs and so on. Last but not least, when the relevant sites of circRNAs are “blocked” due to the occurrence of base pairing, phenomena happen that the ability of circRNAs serving as miRNA “sponge” and translating is hindered.

Actually, several researches have questioned about the mechanism of ceRNA (Thomson and Dinger, 2016): some circRNAs were predicted to be able to bind a certain miRNA and regulate the downstream target genes, while this cannot be well proved through related experiments. On the other hand, the abundance changes of some circRNAs cannot effectively regulate the targeted genes. Based on this phenomenon, we put forward the hypothesis of “open-close effect” in the transition for specific circRNA from normal state to morbid state: for those circRNAs with closed ICBPS, even if they are highly expressed, they may not exert corresponding biological functions; for those circRNAs with similar or even lower expression, they may also play important roles through “opening” relevant ICBPS, and vice versa. In addition, RBPs are also known to be sponged by circRNAs and regulate gene expression. And the existence of ICBPS may affect the regulatory process as in miRNAs (Figure 2C).

## Conclusion and Discussion

The presence of circRNAs is first being discovered in a viroid-infected plant (Sanger et al., 1976). Following evidences approved the critical role of circRNAs in various diseases (Shabaninejad et al., 2019). CircRNAs are ubiquitous, stable, conserved and diverse RNA molecules with a range of activities. They are reported as one of the main players in the regulation of multiple pathways and cellular processes (Yousefi et al., 2020). Some studies also suggested that the expression of circRNAs is dysregulated in virus-infected cells. Virus then uses the cellular mechanism to its advantage (Nahand et al., 2020). Chen et al. (Liu C.X. et al., 2019) first describes the degradation mechanism of circRNAs when cells are infected by virus and exhibited the secondary structures of circRNAs. Structural mapping showed that circRNAs inside cells could form stable secondary structures which contained short imperfect duplexes. Guria et al. (2019) also discussed the possible mechanism of circular RNA biogenesis, its structure and degradation. These studies emphasized the functional importance of the secondary structure within circRNAs. However, the structure and the potential molecular mechanism of circRNAs are still poorly studied.

Here, we provide new ideas and clues for the research of circRNAs. We speculated that circRNA may not be a simple circular structure, which probably contains double-strand structure internally, and the “open” or “close” of the double-strand structures is a sophisticated dynamic process. The hypothesis indicates that circRNA may play roles in the occurrence and development of disease, not necessarily through its aberrant expression change, but also through the “open-close effect” of related sites on the sequence. If a certain circRNA has both oncogenic and tumor suppressor miRNA binding sites, it may selectively “open” or “close” specific miRNA binding sites, consequently leads to different effects. While the main function of circRNAs is exerted through their activity as miRNA sponges, their second-most important function is exerted *via* circRNA-protein interactions. Interacting with regulatory RBPs through their activity as protein sponges, decoys, scaffolds, and recruiters, circRNAs then affect the fate of their target mRNAs (Conn et al., 2015; Yang et al., 2017). Thus, change in circRNAs’ structure due to ICBPS may affect their binding between miRNAs as well as RBPs. In turn, RNA-protein interactions may regulate the synthesis and degradation of circRNAs. The interacted and regulatory relationships induced by ICBPS thereby influence cellular functions and disease processes.

In conclusion, our models may help to provide new ideas and clues for the questions as follows: (1) certain circRNAs cannot be amplified and validated using primers designed according to the design principle; (2) why some circRNAs have no effects on their predicted target miRNAs and RBPs; (3) how can circRNAs be exported from nucleus into cytoplasma (especially for the el-circRNAs); (4) how can circRNAs be degraded; (5) how does m6A regulate circRNA encoding proteins; and (6) this may help to strengthen the functional research of el-circRNAs. This theory may change the existing circRNA research mode, and more importantly, extend the underlying logic of selecting indicators based on circRNA- sequencing or circRNA array.

## Materials and Methods

### Acquisition of Internal Complementary Base-Pairing Sequences

Through the custom python script, we search all the circRNA sequences in circBank database. CircRNAs with two internal completely complementary regions (≥10 nt) are selected. The sequences were acquired and regarded as candidate ICBPS. We used two pointers to represent the start and end of the query sequence and set pointer1 as position 1 bp and pointer2 as position 10 bp at the beginning. If ICBPS was found, pointers2 will move until find the longest ICBPS. if no ICBPS was found pointer1 move 1 bps. Searching will end when the pointer1 move to 20 bp upstream of the end of the circRNA sequence.

### Prediction of miRna Recognition Elements

Through the custom R packages (Pagès et al., 2019), we extracted the ICBPS sequence (≥20 nt). Potential miRNAs that bind to ICBPS were predicted through miRanda (Enright et al., 2003) with relatively strict parameters (free energy ≤ 20, alignment score > 150).

### Assessment of Coding Potential

Predict The coding potential and open reading frame (ORF) of circRNAs were predicted through CPAT (default parameter) (Wang et al., 2013).

### Statistical Analysis

Data analysis was performed using GraphPad Prism7 software (GraphPad Software Inc., La Jolla, CA, United States) and SPSS 20.0 software (SPSS Inc., Chicago, IL, United States). Correlations were analyzed by Pearson’s correlation test. *P* values of <0.05 were considered significant.

## Data Availability Statement

The data that support the findings of this study are openly available in circBank database (http://www.circbank.cn) and circBase (http://www.circbase.org/).

## Author Contributions

HJ, HS, and XD: conceptualization. HS and ZW: Methodology. HS, ZW, and LY: investigation. HS, XD, JZ, and ML: bioinformatical analyses. ZW and HS: writing-original draft. HJ and XD: writing-review and editing. HJ, JL, and LY: supervision and funding acquisition. All authors read and approved the final manuscript.

## Conflict of Interest

ML and JZ were employed by the company Guangzhou Geneseed Biotech Co., Ltd. XD was employed by the department of Bioinformatics, ATCGene Inc. The remaining authors declare that the research was conducted in the absence of any commercial or financial relationships that could be construed as a potential conflict of interest.

## Footnotes

1 http://www.circbank.cn/

## Abbreviations

3′-UTR 3′: untranslated regions
ceRNAs: competing endogenous RNAs
circRNA: circular RNA
circLen: length of circRNAs
CR: complementary ratio
el-circRNAs: extremely long circRNAs
ICBPS: internal complementary base-pairing sequences
IRES: internal ribozyme entry site
m6A: N6-methyladenosine
maxLen: maximum length
medianLen: median length
miRNAs: microRNAs
MREs: miRNA recognition elements
ORF: open reading frame
RBP: RNA-binding protein.
