# Supplementary figures and images for: CircRNA May Not Be “Circular”
Source: Front Genet. 2021 Feb 19;12:633750. doi: 10.3389/fgene.2021.633750 (PMC7934283; doi:10.3389/fgene.2021.633750)

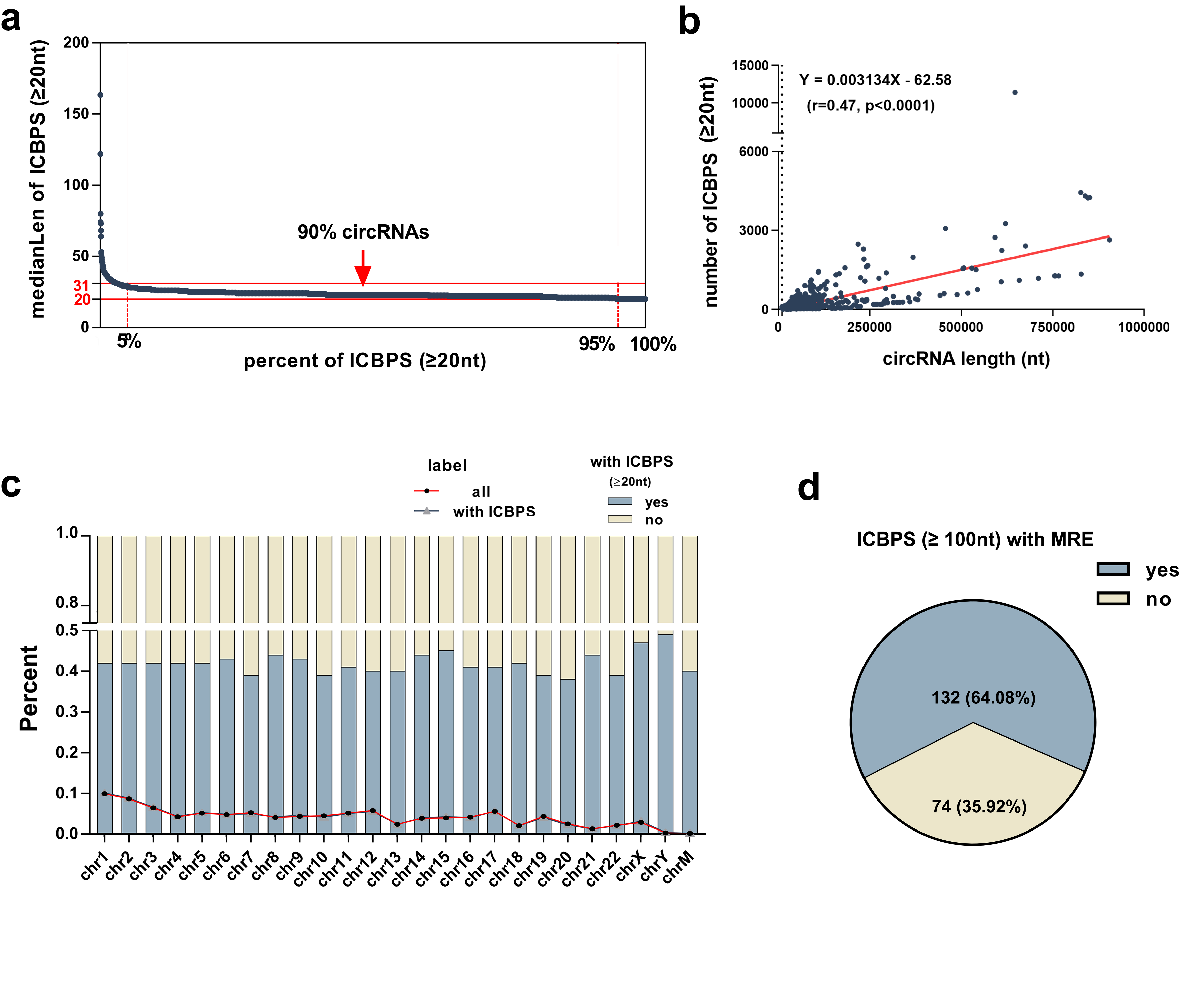

Supplement: Supplementary file 1 [file Image_1.TIF]
