# Supplementary material for: CircRNA May Not Be “Circular”
Source: Front Genet. 2021 Feb 19;12:633750. doi: 10.3389/fgene.2021.633750 (PMC7934283; doi:10.3389/fgene.2021.633750)
Supplement: Supplementary file 3 [file Data_Sheet_1.PDF]

Supplementary Data S1

Information of the instanced circRNA

circBase ID: hsa_circ_0000527

circBank ID: hsa_circFAM158A_001

Position: chr14: 24610097-24616430

Length: 6071

RNA sequence:

CTGGACCAGTCCGGTACGCTCCGGGCCTCACCGAGGAAGTCCCACCCTGC
AGGGGAACCGACTCTGGCCGGCCCTAACCCCTCTCGAGCGAGTCCCGCCC
CATCTCCGGTCTGTCCTGAAGGCAGAGGGAAGACCCTCACCCAACCCCTG
CCCGCTCTCTTCTAAGAGCTGCTAGTCCTTCTCCACCCCAGGCGCTCCCTCT
GGCTGCCCCTCCCACTCGCTCTGATCTCTCTCCGTCCGCTACTTGCGGTCGA
GCCTTCTTGGGGGAGCCCCGCCACTGAATACCCCCTTTGCCATACTCCCAC
CTCTCTTACCCCAACCCTGGACTTCCAGTTGGCCCTAGCTATAAGCCCCGCC
CCTCTCCGAGGGACAGCCCCCGCTCCAGTCTCCGCGGTCCCACTTACCCTA
GCCCCTCCCTCGGGCAGCCCCTGGACTGTGACCCAGCCAGCACCGCCTCT
CCTAGTGGCAACTGGCTGGGAATGGCTGAGGGGCTACCTACTGCTTGCGGC
CTGCCAGCGGGAGGAGGGGGGCGGAAAGAAGAAAGGGGGCGGGGTTGA
GGGGGGCGGGCCTGGACCTGGGGAGTGAAAGCGAAAGCCCGGGCGACTA
GCCGGGAGACCAGAGATCTAGCGACTGAAGCAGCATGGCCAAGCCGTGTG
GGGTGCGCCTGAGCGGGGAAGCCCGCAAACAGGTAATGGGGAATCGGAG
GCCAGGAAGGGGTCATAGAACCCTCTTGGGTGGAGGCCAAGCTGGGTGGT
TCAGGGGACCAAGCAGTGCCAGAGAACTCCCCCAATCTCGCTAGTGCCTT
CACTTCTCGGGGATTCCCAGATTCCTGCCTTCTGACTGCAGTGAGCAGGAG
CTTCCAGGGAAAGCTAGTTTGTGGTGAGAAAGGCCTGAGCCACAGTTCGA
GGGCACCCTGTTGTGGGTGGAAGCTTTTGACAACCCAGACCCTTAACCAA
CATCCGGGTACTGCTCTCTGTGTAGGTGGAGGTCTTCAGACAGAATCTTTT
CCAGGAGGTAAGTCTCTGGATTTGAGCGATTTGACCCAGAATCTTGGACCA
ATACTTGAGGATAACAGCCTCTCAGAATGACCCCCCCAAGTCACCCTAGAC

TTAACCCTACTTCCCTTACCAAGCCTTCACCCAAAGTAACCCCGGAATTATT
GATAGCTACCTTTAATCTGACCTTTCTTATGACTCTCCCGACTTCTGGCACTT
CTCCCGTCTTTTGATGTGGAGGAAAAAAACCATGGCTTCCAAAGCAGGAAT
ATCACAGTCCTAACCTTCGTTCTGCCATTTACCAGCTTGGGCAAGTCCTTTC
ATTTTCAGGTCTTCAGTTTCCTCATTTGCAAGTCTTGGACCCAGTGATTTCC
AAAGGCCCTTCCAGTTCCATTTGTCAGACATCTCATTGAGCATATTCTGTGA
TTCCCCTCCCTTGCAATGTAACCCACAGGCTGAGGAATTCCTCTACAGATTC
TTGCCACAGAAAATCATATACCTGAATCAGCTCTTGCAAGTGAGTAGTGTG
GGCCCCTTCTGCCCCATCCTATGTCCTCTATCCCCTTTGCCCACCCTCTCTAG
CTAACAACTGGATTCAGCATGCAGAGAATAGTTTCAAATAGTGGATTAGGA
ACATTAGGATGGCGTGAGGCATGAGCCTAGGTTGGGAGGCAGAGTGCAAG
TGGGATGTATGGAGGGAAGGCTGTGACCTAGGATGAAGGGAAGAGGTTGG
GCAGCCCTTGACAGTGAGCTCTGTGCAGGAGGACTCCCTCAATGTGGCTG
ACTTGACTTCCCTCCGGGCCCCACTGGACATCCCCATCCCAGACCCTCCAC
CCAAGGATGATGAGGTGAGG[CACTCAGGGTGC**AGGACTTGGACTATAAA**
CCCAATGGAGAAGATAGCCCTTCAACCTCTGTGACTTTTCTAAAGCTA
CTTTCCCCCCTTTTTGCCTTAGATGGAAACAGATAAGCAGGAGAAGAA
AGAAGGTAAGAGAGATAAGAGGTXXXXXXXXXXXXXXXXXXXXXXXXX
XXXXXGGATTCAGGCCCTTTCTCATCCAGTAGTCAATGTGCCATCTCCCCTT
CCCTAGTCACCTCTTATCTCA**CTTACCTTCTTTCTTCTCCTGCTTATCTGT**
TTCCATCTAAGGCAAAAAGGGGGGAAAGTAGCTTTAGAAAAGTCACAG
AGGTTGAAGGGCTATCTTCTCCATTGGGTTTATAGTCCAAGTCCTTCAC
CCTGAGTGCCTAGGGTG]GGGATTGTTACATGCCATGGCAGCCATGCAGATA
ACCTGTATTTTGGAATGTAACAGTATTATTTTCATCCAGAGGTCTCAGGAAT
TATTGGAAGAGATGGCATTTGCACAGAGCCCTTATGGGAATAATCAGGCTTT
GGATACACAAGGGGAAAGTTCTGTGGTCAACATCAGGAATGAAGAAAGAA
AGAATCCTCATCAGTTCAGGAAAAGCCTAGAAGGAGAATAAGGTTTCACT
GGGAGAACTATGGAGAACTACGGATGAATGATTGGACTCCCCCCACCCTTT
TTTTTTTTTTTTTGAGATGGAGTCTTGCTCTGTTGCCCAGGCTGGAGTGCAA
TGGCACGATCTCAGCTTACTGCTACCTCCGTCTCCCGGGTTCAAGCAATTCT

CCTGCCTCAGCCTCCCGAGTAGCTGGAATTACAGGCATGTGCCACCACACC

TGGCTAATTTTTTTGTATTTTTAATAGAGACGGGGTTTCGCCATGTTGGCCA

GGCTGGTCTCGAACTCCTGACCTCAGGCGATCCACCCACCTCGGCCTCCCA

AAGTGCTGGGATTACAAGGCGTGAGCCACCGCGCCGGGCCTGGACTCCCT

CCTTAAGGGCTAGGAGGGGAAAAAATAATGCTTCCAAGGCACTGTGGTGA

AGGAAATACATCGCAGGACATTATGGGAAGGATGAATCTGTAAGTTTTTCTT

ACTGTCCTCCCCCATATCCCCTTCAGCCAATCACTGTCTTTATCTCTGCTTTT

CAAGATTCAGCCCTGGGCTGGTGATAGAGACAAGGGTTCCTTTGCTGTGAT

CTTGCTGTAAACTCCATGGGATTGTTCTTTAATTCAGGGCCTTATCCTGTCAT

AAAATATGAGATAAGAATGTGCTGCGAAGCACTAGACTGGCTGGAAGCCTG

CCCCCATACATATCTCATTAAATCCTTAAAGGAACACGGGCAAAAGATAAAC

CAAGGGCAAGAAAACCTGCAGTCGCGAAGGCAACACGGCCCATTAGTAAG

TGACAGAGCCGATCTAGAGTACAGAGCCACTCAACCCATAATAGGCGCAA

GGCGGTCTGCTTTGCCCGCAGCGCTCCGGAACACCCGGCTACGGGATAGC

GATCCATCCTTGGGGCTCGGCGGAGCGAGTGGTAGTGGGCAACACAGGTA

ACAGGCAAAGCCAACCACCAACCGAGAGCGGAAGTCGGGGAACCTAGCC

AATAAGAGTGCGGGCAATTTTCTGTGCTTCACAATGGTGGGTTCTTTCCAG

TCCGCTGGAGACAAAGGCGGGGCAACGTTGGGGCGCGGCCGCTGATTGGA

CAAATTGAAAGCCTTTAGCCCTATCAAGGAGCACCACGTGGGCGGGACTC

CAGGAGCCAGTTCTGCAGGGAGCTGCAGTTTGGTCTGTCTCCCAGGCAGG

CGCAGAGTCGCGGCCGCCAGCTAGGGGCGCGGGAAGGCGGGGCTCGGAT

GCAATCGGGACCTCCTCCTGGACTGGGCCGGGGGCGGACTCCGGGACCCA

GGGCGCCGGGAGCCGGCGGGCTACCTGCGAGTCGAGTTAGCGTTGTCGCC

GAACCGAAGCCTCGCTCGCCATGGGGGAGGTGGAGATCTCGGCCCTGGCC

TACGTGAAGATGTGCCTGCATGCTGCCCGGTACCCACACGCCGCAGTCAAC

GGGCTGTTTTTGGCGCCAGCGCCGCGGTCTGGAGAATGCCTGTGCCTCACC

GACTGTGTGCCCCTCTTCCACAGCCACCTGGCCCTGTCCGTCATGTTGGAG

GTCGCCCTCAACCAGGTGGATGTGTGGGGAGCACAGGCCGGTCTGGTGGT

GGCTGGTTACTACCATGCCAATGCAGCTGTGAACGATCAGAG

ICBPS is noted with red color and XXXXXX indicate an omission in the sequence

Supplementary Figure S1

Figure S1. (a) The median length of 6,155 circRNAs' ICBPS. (b) The correlation analysis between the number of ICBPS (⩾20 nt) and the total length of circRNAs (> 10,000 nt). (c) The percentage of circRNAs (with ICBPS ≥ 20 nt or not) that transcribed from different chromosome origins. The polyline graph shows the number distribution of circRNAs in different types. (d) The distribution of circRNAs (with ICBPS ≥100 nt) with or without MRE.
